# Alleviating effects of KGC19b on periodontal inflammation through anti-inflammatory and induction of osteogenic differentiation in *in vitro* and *in vivo* experimental models

**DOI:** 10.1016/j.jgr.2026.101014

**Published:** 2026-02-21

**Authors:** Da Eun Lee, Eun-Nam Kim, Nguyen Minh Trang, Jong Han Kim, Gi-Bang Koo, You Hee Jung, Gil-Saeng Jeong

**Affiliations:** a College of Pharmacy, Chungnam National University, Daejeon, 34134, Republic of Korea; b Laboratory of Efficacy Research, Korea Ginseng Corporation, Gyeonggi-do, 13480, Republic of Korea; c Laboratory of Standards and Fundamental Research, Korea Ginseng Corporation, Gyeonggi-do, 13480, Republic of Korea

**Keywords:** Anti-inflammation, Human periodontal ligament, KGC19b, Osteogenic induction, Periodontitis, *Porphyromonas gingivalis*

## Abstract

**Background:**

*Panax ginseng* Meyer of the Araliaceae family have various biological activities and pharmacological actions such as anticancer, immunomodulation, and anti-inflammatory. In many studies on ginseng, studies on ginseng fruit are still new, and the effect and potential of ginseng fruit on periodontitis in HPDL cells have been investigated *in vitro* and *in vivo.*

**Methods:**

HPDL cells were stimulated with PG-LPS for 12 h to evaluate the effects of KGC19b (50–200 μg/mL) on periodontitis. Cell viability, pro-inflammatory markers, reactive oxygen species (ROS), and osteogenesis-related markers were assessed. In the rat model, periodontitis was induced using ligature and PG-LPS, and KGC19b was administered orally for 6 days. The effects of KGC19b were evaluated by micro-CT analysis and H&E staining.

**Results:**

KGC19b reduced pro-inflammatory cytokine expression, restored osteogenic markers, enhanced antioxidant enzyme activity, and decreased ROS in HPDL cells. In the rat model, KGC19b alleviated periodontal inflammation and prevented bone mass loss.

**Conclusion:**

In this study, KGC19b shows the possibility of improving and treating periodontitis through anti-inflammatory, osteogenesis, and antibacterial effects.

## Graphical abstract

**Figure:**
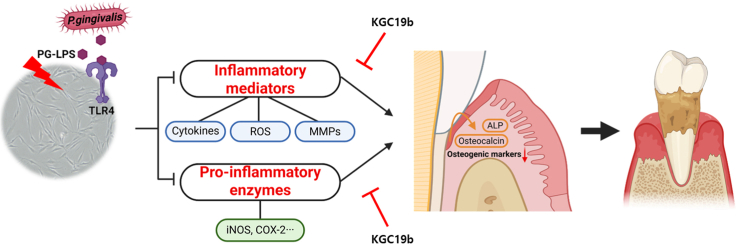

## 1 Introduction

Periodontitis is a chronic inflammatory disease caused by bacterial plaque that damages tooth-supporting tissues and alveolar bone, ultimately leading to tooth loss [1,2]. It affects 10–15% of the global population and primarily occurs in adults, though it can also affect children [[3], [4], [5], [6], [7]]. Current treatment options, including antibiotics and non-surgical or surgical periodontal therapy, help control infection and inflammation but are limited in promoting tissue regeneration [8,9]. Consequently, there is increasing interest in natural products with proven efficacy and safety as alternative therapeutic strategies [10].

*Porphyromonas gingivalis* is a Gram-negative anaerobic bacterium residing in the oral cavity, and dysbiosis of the oral microbiota induced by *P. gingivalis* plays a critical role in the development of periodontal disease [[11], [12], [13]]. Its pathogenicity is largely mediated by lipopolysaccharide (PG-LPS), a key virulence factor that disrupts host immune homeostasis [14]. Recent studies have shown that TLR4 plays a pivotal role in inflammatory responses by regulating immune cell activation and differentiation [15]. In addition, binding of PG-LPS to TLR4 activates downstream signaling pathways that promote pro-inflammatory cytokine production and disrupt osteogenic differentiation, thereby accelerating periodontal tissue destruction [[16], [17], [18], [19], [20], [21], [22], [23], [24], [25], [26], [27], [28]]. Furthermore, activation of TLR4 has been shown to induce the accumulation of reactive oxygen species (ROS), thereby exacerbating oxidative stress within the microenvironment [29]. Collectively, these findings suggest that the TLR4-mediated inflammatory and oxidative stress axis plays an important role in the pathogenesis of periodontitis.

Among the *in vivo* models, the *Porphyromonas gingivalis* lipopolysaccharide (PG-LPS) injection model and the ligature-induced periodontitis model are widely used [27,28,[30], [31], [32]]. Among *in vivo* approaches, the PG-LPS injection model induces acute periodontal inflammation [33], while the ligature-induced model mimics chronic disease progression through bacterial accumulation and mechanical irritation [[34], [35], [36], [37], [38], [39]]. Previous studies have reported the beneficial effects of ginseng extracts and ginsenosides in these models [[40], [41], [42]]. However, few studies have applied both models to comprehensively evaluate anti-inflammatory and bone-regenerative effects [28,36].

Ginseng (*Panax ginseng* Meyer), widely cultivated in Northeast Asia, exhibits diverse pharmacological activities such as antioxidant, anti-inflammatory, and immunomodulatory effects [[43], [44], [45], [46]]. Recent studies have focused on its roles in bone metabolism and periodontal disease, effects mainly attributed to ginsenosides [47]. Ginseng fruits contain higher levels of specific ginsenosides, including Re, Ra8, and Rf, than ginseng roots [48]. Although the individual effects of these ginsenosides on periodontitis have been documented [49], the comprehensive therapeutic potential of whole ginseng fruit extract remains relatively unexplored. In line with the need to investigate fruit-derived compounds, we previously demonstrated that these ginsenosides exert protective effects against periodontitis by modulating the HO-1/EGFR signaling axis [50,51]. However, it remains unclear whether ginseng fruit–derived compounds can modulate the PG-LPS–Toll-like receptor 4 (TLR4)–mediated signaling pathway, which is recognized as a major etiological mechanism in the development of periodontitis. Therefore, the objective of this study was to determine whether KGC19b, a standardized extract derived from ginseng fruit, can attenuate PG-LPS–induced periodontitis by suppressing TLR4-mediated signaling and thereby preserve osteogenic function under inflammatory conditions, using both *in vitro* and *in vivo* models.

## 2 Materials and methods

### 2.1 Materials

Cell culture was performed using α-MEM (LM008-02) supplemented with FBS (10082147), along with penicillin/streptomycin and trypsin-EDTA (15400054), all sourced from Gibco, Grand Island, NY, USA. For cell viability assessment, MTT (M2128) was procured from Amresco Inc, Cleveland, OH, USA. Bacterial lipopolysaccharide from *Porphyromonas gingivalis* (PG-LPS, tlrl-pglps) was sourced from InvivoGen, San Diego, CA, USA. Recombinant cytokines including TNF-α (PRTA00), IL-6 (PR6000B), and IL-1β (PRLB00) were supplied by R & D Systems, Minneapolis, MN, USA. Primary antibodies against iNOS (sc-7271) and COX-2 (sc-166475) were obtained from Santa Cruz Biotechnology Inc., Dallas, TX, USA. Secondary antibodies for western blotting included anti-rabbit (7074P2) and anti-mouse (7076P2) from the same supplier. The detection system consisted of Hybond ECL nitrocellulose membranes (Amersham Pharmacia Biotech Inc., Piscataway, NJ, USA) and western blotting reagents from Advansta Inc., Santa Clara, CA, USA. Alizarin Red S staining solution was supplied by Sigma-Aldrich, St. Louis, MO, USA (TMS-008-C). RNA extraction was performed using TRIzol reagent (TS200-001) from Bio Science Technology, Seoul, Korea. The reference standard ginsenoside Re was purchased from Chromadex Co., Irvine, CA, USA. HPLC-grade acetonitrile and methanol were supplied by Merck, Darmstadt, Germany. Purified water was prepared using a Milli-Q water purification system (ZEQ7008T0C, Millipore, Bedford, MA, USA), with resistance confirmed at 18 MΩ before experimental use. All chemical reagents met guaranteed reagent grade specifications.

### 2.2 Extract preparation and standardization

The sampling was conducted on private ginseng farm (Chungbuk province, Republic of Korea), and the farm owner collected in July 2021 and identified by JongHan Kim (Natural Product Efficacy Research Institute, Korea Ginseng Corporation inc., Republic of Korea). A voucher specimen was deposited in the Material Development team of the Korea Ginseng Corporation (KGCRM20210726001, Gwacheon, Republic of Korea). A total of 1,900 kg of ginseng fruits were harvested and thoroughly washed with water, then separated into pulp and seeds. The separated pulp was juiced, and the juice was extracted in a tank at 85 °C for 3 h without adding any additional purified water. The extract was then filtered and concentrated, and 10% dextrin was added to the concentrate before spray drying to obtain 73 kg of extract powder. The Korean ginseng fruits used in the study were grown and purchased from a contract farm managed by Korea Ginseng Corporation (KGC, Republic of Korea), and the sampling of the samples was also provided with the permission of the landowner.

### 2.3 Preparation of HPLC sample and liquid chromatographic

KGC19b sample (0.5 g) was placed into a volumetric flask and extracted with 50 mL of 70% MeOH. Ultrasonic extraction was carried out for 30 min, followed by cooling to room temperature. The extracted solution was filtered through a 0.2 μm PVDF syringe filter (Whatman, UK) prior to HPLC analysis. Chromatographic analysis was conducted using a Waters Alliance HPLC system (Waters Co., Milford, MA) configured with a binary solvent delivery pump, autosampler, and photodiode array (PDA) detector. Signal detection was performed at 203 nm wavelength, with data acquisition and processing managed through Empower 2 software. Separation was achieved using a reversed-phase Discovery C18 column (5 μm particle size, 4.6 × 250 mm, Supelco). Temperature control was set to 30 °C for the column and 25 °C for the autosampler tray. The mobile phase system employed acetonitrile (solvent A) and water (solvent B) delivered at 1.0 ml/min flow rate. The gradient elution program was programmed as follows: 0-5 min (20% A), 20 min (23% A), 25 min (30% A), 45 min (40% A), 55 min (50% A), 65 min (50% A), and 70-75 min (20% A) with sequential adjustments.

### 2.4 Cell culture and cell viability

**Fig. 1:**
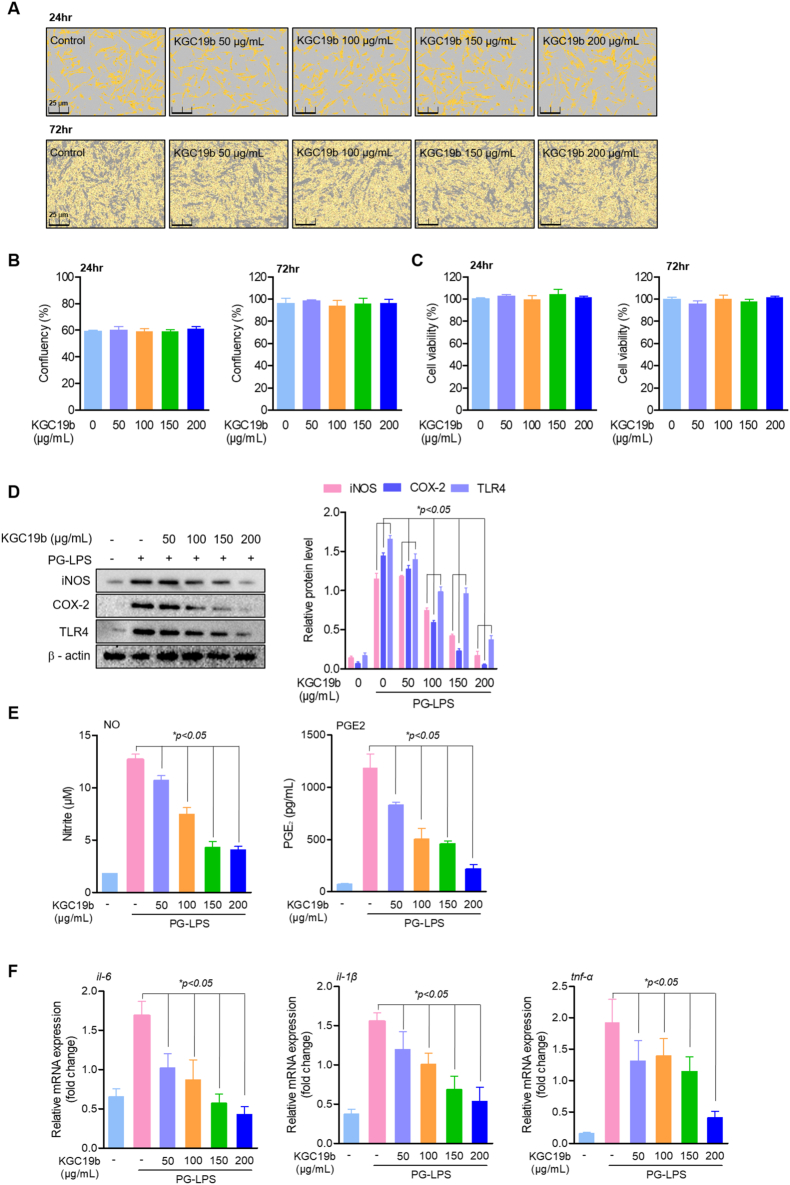
Effects of KGC19b on cell viability and anti-inflammatory responses in HPDL cells. (A, C) HPDL cell confluency was assessed using the Incucyte® Live-Cell imaging system after treatment with KGC19b (0–200 μg/mL) for 24-72 h. (B) Cell viability was evaluated by MTT assay after treating HPDL cells (5 × 10^3^ cells/mL) with KGC19b at the indicated concentrations for 24-72 h. (D) HPDL cells were pretreated with KGC19b (50–200 μg/mL) for 6 h, followed by stimulation with or without PG-LPS (1 μg/mL) for an additional 6 h. The expression levels of TLR4, COX-2, and iNOS were analyzed by western blotting. (E) The production of inflammatory mediators NO and PGE2 was measured in the culture supernatants. (F) mRNA expression levels of pro-inflammatory cytokines *il-6, il-1β,* and *tnf-α* were quantified by real-time PCR. Data are presented as mean ± SD (*n* = 3). *∗p* < 0.05 vs. PG-LPS-treated group.

The human periodontal ligament (HPDL) cells employed in this investigation were sourced from Kyungpook National University School of Dentistry, Daegu, South Korea, with approval obtained from the Kyungpook National University Institutional Review Board (KNU 2017-78). HPDL cells were isolated according to a validated protocol reported previously [52,53]. Cells were isolated by harvesting periodontal ligament tissue from donor third molars, which underwent digestion with type I collagenase before being processed into single-cell suspensions for cultivation. Cell maintenance was performed using α-MEM medium containing 10% (*v/v*) FBS and 1% penicillin/streptomycin (Cat. No. SV30010, Gibco BRL, Grand Island, NY, USA) under standard incubation conditions at 37 °C. To assess KGC19b cytotoxicity against HPDL cells, both cell counting analysis and MTT viability assays were conducted. HPDL cells were seeded into 96-well plates at 5 × 10^4^ cells/mL density and maintained in a humidified environment (37 °C, 5% CO_2_) for 24 h. Cell confluency monitoring was carried out using the Incucyte® Live-Cell Analysis System (Göttingen, Germany). The isolated cells exhibited a uniform spindle-shaped morphology, strong substrate adhesion, and contact-inhibited growth pattern, characteristic of HPDL-derived mesenchymal cells, throughout the early passages (P3–P5). Morphological confirmation of this using the IncuCyte Live-Cell system is shown in Fig. 1A. The MTT assay protocol involved adding 50 μL MTT solution to cultured cells, followed by 3-h incubation. Following medium removal, 200 μL of DMSO (Cat. No. 1380) was introduced to each well. Optical density measurements were subsequently obtained at 540 nm wavelength using a microplate reader (Tecan Trading AG, Männedorf, Switzerland).

### 2.5 Western blot analysis

HPDL cells were seeded at 5 × 10^4^ cell/mL density in 6-well plates and maintained under humidified conditions (37 °C, 5% CO_2_) for 24 h. Following this incubation period, cells underwent treatment with KGC19b at various concentrations ranging from 50 to 200 μg/mL for 6 h, subsequently followed by PG-LPS stimulation for 12 h. Cell lysis was performed using RIPA buffer (Sigma-Aldrich, USA), and the resulting lysates underwent centrifugation at 23,000×*g* for 15 min. Bradford assay was employed to quantify protein concentrations in the supernatant, with 20 μg protein samples subjected to electrophoresis on 12% SDS-PAGE gels. Following electrophoretic separation, proteins were transferred onto PVDF membranes, which were subsequently blocked using 5% non-fat dried milk for 1 h at room temperature. Primary antibody incubation was carried out at 4 °C for 24 h, followed by secondary antibody treatment for 2 h. Detection of antibody-bound activated proteins was accomplished using the ECL Western Blotting Detection Imaging System [54].

### 2.6 Real-time PCR

Following treatment with all test concentrations for 6 h, samples underwent PG-LPS stimulation for an additional 6 h. Total RNA extraction was performed using TRIzol/chloroform reagent, and the extracted cDNA was subsequently quantified via nanodrop measurement. Target gene amplification and analysis were conducted using the Prime Script-RT reagent kit with SYBR Premix Ex Taq. Normalization of cycle threshold (Ct) values for each target gene was achieved using *gapdh* as the housekeeping gene reference [55]. All primer sequences utilized in this investigation are detailed in Table S1 and were obtained from Bioneer, Daejeon, Korea.

### 2.7 Mineralization assay

For assessment of KGC19b effects on HPDL cell osteogenic differentiation, cells were plated in 24-well plates at 1 × 10^4^ cells/mL concentration and maintained under humidified conditions (37 °C, 5% CO_2_) for 24 h, followed by 1 h of initial culture. The culture medium was then substituted with osteoblast differentiation medium (ODM) supplemented with 50 μg/mL ascorbic acid, 0.1 μM dexamethasone, and 10 mM β-glycerophosphate. Treatment with various KGC19b concentrations (50-200 μg/mL) was applied for 3 h incubation. The samples underwent 7-day culture with simultaneous PG-LPS treatment, after which 14-day cultivation was continued. Cell fixation was achieved using 4% paraformaldehyde treatment for 30 min, then stained with 0.1% Alizarin Red S (Sigma-Aldrich, St. Louis, MO, USA) at pH 4.3 for 1 h at room temperature. Deionized water was used to wash the samples afterward. Documentation of staining results was performed through optical microscope photography (Nicon Japan). Quantitative measurement of the dissolved precipitate was conducted using a microplate reader (M1000 Pro, TECAN, Männedorf, Switzerland) at 570 nm wavelength [56].

### 2.8 Intracellular ROS measurement

HPDL cells were seeded at 5 × 10^4^ cell/mL density in 6-well plates and maintained under humidified conditions (37 °C, 5% CO2) for 24 h. Treatment with various concentrations (50-200 μg/mL) was applied for 6 h, followed by PG-LPS stimulation for 24 h. Cells underwent incubation with 2',7'-dichlorodihydrofluorescein diacetate (DCF-DA) (Cat. No. 35845, Sigma-Aldrich Fine Chemicals; St. Louis, MO, USA) for 30 min at 37 °C. Following PBS washing, ROS level changes were measured over 30 min using the Incucyte® Live-Cell Analysis System. The ROS level (% of control) represents the relative fluorescence intensity of each treatment group compared to the control group. The fold change in ROS was calculated using the fluorescence value detected when the fluorescence intensity of the control group was set to 100% [57].

### 2.9 Determination of minimum inhibitory concentration (MIC) and minimum bactericidal concentration (MBC)

*P. gingivalis* (KCTC 5352) was sourced from the Biological Resources Center of the Jeonbuk Branch of the Korea Research Institute of Bioscience and Biotechnology. Bacterial culture was performed in TSA hemin menadione medium at 1 × 10^2^ colony forming units [CFU]/mL concentration for 24 h, with KGC19b concentrations of 10, 20, 40, 80, 160, 200, 400, 800 μg/mL under humidified atmosphere (37 °C, 5% CO_2_). MIC analysis involved absorbance measurement at 600 nm wavelength using a microplate reader. Following MIC determination, MBC analysis was conducted using concentration groups of 140, 150, 160, 200, and 400 μg/mL, with absorbance analyzed at 600 nm wavelength via microplate reader [58].

### 2.10 Animal

Male 8-week-old Sprague–Dawley rats were purchased from Samtako Inc. (Osan, Korea) for experimental use. All animal procedures were performed according to the "Animal Use Guidelines" established by the Chungnam National University Institutional Animal Care and Use Committee. Approval for experimental methods and procedures was obtained from the Chungnam National University Institutional Animal Care and Use Committee (No. 202203-CNU-063), with experiments conducted at the Chungnam National University Joint Animal Research Center. Animal euthanasia was performed at experiment completion following Institutional Animal Care and Use Committee guidelines of Chungnam National University (Daejeon, Korea). Carbon dioxide (CO_2_) inhalation was used for euthanasia to ensure complete cessation of vital functions. Death confirmation involved assessment of heartbeat absence, respiratory arrest, and corneal reflex loss. These procedures ensured ethical standards compliance and proper animal handling throughout the study.

### 2.11 Ligature-induced periodontitis model

The Ligature-Induced Periodontitis Model employed both oral administration and topical application of KGC19b. For oral administration studies, 8-week-old male SD-Rats were divided into groups (each group, n = 6, total of 36, each group: control group, group induced only by ligature, ligature + KGC19b 50 mg/kg, ligature + KGC19b 100 mg/kg, ligature + KGC19b 150 mg/kg, ligature + KGC19b 200 mg/kg). Following CO_2_ inhalation anesthesia, the right maxillary first molar was secured and ligated using non-absorbable silk sutures. Daily inspection of ligature sites was conducted to monitor periodontal inflammation development, with free diet therapy provided. On day 6 post-induction, the non-wet silk ligature was removed along with first molar extraction. After confirming the presence of an alveolar bone defect at the site of the extracted first molar, KGC19b was orally administered at the indicated doses for 14 days. For local treatment, KGC19b at the indicated concentrations was applied directly to the extraction socket, and the upper portion of the socket was covered with Pluronic® F-127 (Cat. No. P2443, Sigma-Aldrich, St. Louis, MO) to maintain local drug retention [59,60].

### 2.12 PG-LPS-induced periodontitis model

Before inducing PG-LPS periodontitis, 8-week SD-Rats received oral administration of KGC19b at doses of 50, 100, 150, and 200 mg/kg for 14 days (each group, n = 6, total of 36, each group: control group, group induced only PG-LPS, PG-LPS + KGC19b 50 mg/kg, PG-LPS + KGC19b 100 mg/kg, PG-LPS + KGC19b 150 mg/kg, PG-LPS + KGC19b 200 mg/kg). General anesthesia was subsequently administered for PG-LPS injection between first and second mandibular molars. Periodontitis induction was carried out for 6 days, with concurrent oral KGC19b administration for 14 days. Upon experiment completion, KGC19b effects on inflammatory infiltration and cytokine regulation were assessed through H&E staining of periodontal tissue regions where PG-LPS-induced inflammation occurred [59,60].

### 2.13 Serum cytokines (IL-6, IL-1β, TNF-α) ELISA assay

Blood sample collection from rat veins was performed following experiment completion. Sample centrifugation and serum separation were conducted at 4 °C low temperature. Separated blood crystals underwent sealing and storage at −20 °C until analysis. Each antibody was cultured in 96-well plates for cytokine analysis, followed by washing buffer treatment of each well. Diluent addition and room temperature incubation for 1 h preceded sample processing and 3-h room temperature incubation. Secondary washing was performed, followed by Streptavidin-HRP B solution treatment and 30 min incubation. Substrate solution was added to well plates, incubated at room temperature with light protection, then analyzed at 540 nm wavelength using a plate reader (Tecan Trading AG, Männedorf, Switzerland).

### 2.14 Micro-CT imaging and analysis

Following experiment completion, sacrificed experimental animals underwent maxillary skull micro-CT imaging (Quantum FX micro-CT, PerkinElmer, MA, USA). Periodontal tissue morphology and bone mineral density (BMD) measurements of maxillary skulls were conducted under the following conditions: tube voltage (90 kVp), tube current (160 uA), imaging time (180 s), FOV (field of view, 5 mm), and pixel size (10 μM). Micro-CT image data were utilized for bone density measurements. Scanned images were loaded into CTAn for analysis based on threshold values obtained from scanned images. Region of interest (ROI) was established from the alveolar bone root of the cement-enamel joint, followed by interpolation method analysis. Data expression used mean ± standard deviation (SD), with data significance evaluated through one-way analysis of variance (ANOVA) using SPSS Statistics (Armonk, NY, USA).

### 2.15 Measurements of CEJ-ABC

The analysis area of interest was established as the region from the cement-enamel junction (CEJ) between second and third molars in the fixed maxilla extending to the alveolar bone ridge (ABC). Measurement sites included buccal sulcus, palatal palate, mesial buccal, distal buccal, mesial palatal, and distal palatal linear areas. Distance measurements were taken between the most distal roots of second and third molars and the occlusal surface of teeth.

### 2.16 Histological staining

Periodontal tissue inflammatory infiltration was confirmed using periodontal tissue removed from maxillary skulls obtained from sacrificed experimental animals at experiment end. Tissue fixation was performed with 10% formalin, followed by paraffin embedding, 5 μm sectioning, and slide fixation for 3 weeks. H&E staining was conducted on sectioned tissues, with photography performed using an Olympus IX microscope 71-F3 2 PH (Tokyo, Japan). Identical regions of interest were photographed for all samples. White soft tissue areas and infiltrated tissues of control, periodontitis-induced, and treatment groups were distinguished and expressed as percentages.

### 2.17 Statistical analysis

For *in vitro* studies, the results were obtained from three independent replicates, and for *in vivo* studies, from six experimental animals per group. Each result is presented as mean ± SD. Group statistical differences were evaluated using one-way ANOVA with Tukey's test or Student's t-test. SPSS Statistics 19.0 software (Armonk, NY, USA) was used for analyses, with p-values below 0.05 considered statistically significant.

## 3 Results

### 3.1 Effects of KGC19b on cell viability and inflammatory responses in PG-LPS-induced HPDL cells

KGC19b, a ginseng fruit-derived extract containing ginsenoside Re as its major component (Fig. S1 and S2), showed no cytotoxicity in HPDL cells at concentrations up to 200 μg/mL over 72 h, as confirmed by confluence and cell viability assays (Fig. 1A–C). Based on these results, concentrations of 50–200 μg/mL were selected for subsequent mechanistic analyses. Upon stimulation with PG-LPS (1 μg/mL), HPDL cells exhibited a marked induction of inflammatory signaling. Treatment with KGC19b for 6 h resulted in a significant and dose-dependent reduction in the protein expression levels of TLR4, iNOS, and COX-2 (Fig. 1D). Consistently, the production of downstream inflammatory mediators, NO and PGE_2_, was significantly suppressed in a concentration-dependent manner (Fig. 1E). Furthermore, qPCR analysis demonstrated that KGC19b significantly downregulated the mRNA expression of pro-inflammatory cytokines *il-6*, *il-1β*, and *tnf-α* (Fig. 1F). Taken together, these results demonstrate that KGC19b effectively suppresses PG-LPS–induced inflammatory responses in HPDL cells by inhibiting upstream TLR4 associated inflammatory signaling.

### 3.2 KGC19b promotes osteogenic differentiation in HPDL cells

**Fig. 2:**
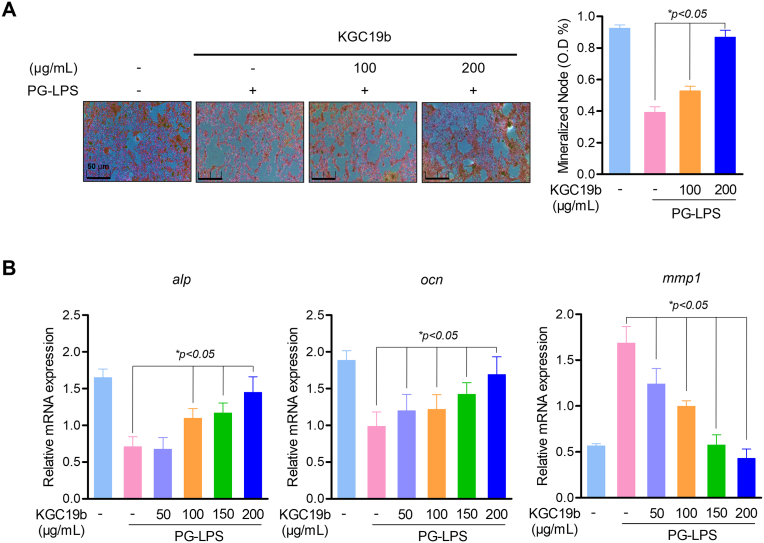
Osteogenic induction effect of KGC19b in HPDL. (A) The HPDL cells were pretreated at the indicated concentration of KGC19b (50-200 μg/mL) for 6 h and then incubated with PG-LPS for 14 days. (B) The level of osteoblast differentiation gene *ocn, alp* and *mmp-*1 mRNA were measured by real-time PCR analysis. Data are expressed as mean ± SD (*n* = 3). *∗p* < 0.05, versus the only PG-LPS treated group.

Given that sustained inflammation is known to impair osteoblast function, this study next investigated whether KGC19b could restore osteogenic differentiation under PG-LPS induced inflammatory conditions. Alizarin Red S staining revealed that PG-LPS markedly inhibited mineralized nodule formation in HPDL cells, whereas treatment with KGC19b significantly and dose-dependently restored mineral deposition (Fig. 2A). Consistent with these observations, KGC19b significantly increased the expression of key osteogenic markers, Osteocalcin (OCN) and Alkaline Phosphatase (ALP), while concurrently reducing the expression of the matrix-degrading enzyme Matrix Metalloproteinase 1 (MMP-1) (Fig. 2B). These results indicate that KGC19b not only counteracts inflammation but also functionally rescues osteogenic differentiation that is suppressed by PG-LPS. Importantly, these findings directly link suppression of TLR4 driven inflammation with the preservation of osteoblast function, suggesting that inflammatory regulation by KGC19b translates into enhanced periodontal tissue regenerative potential.

### 3.3 KGC19b attenuates oxidative stress in PG-LPS–induced HPDL cells

**Fig. 3:**
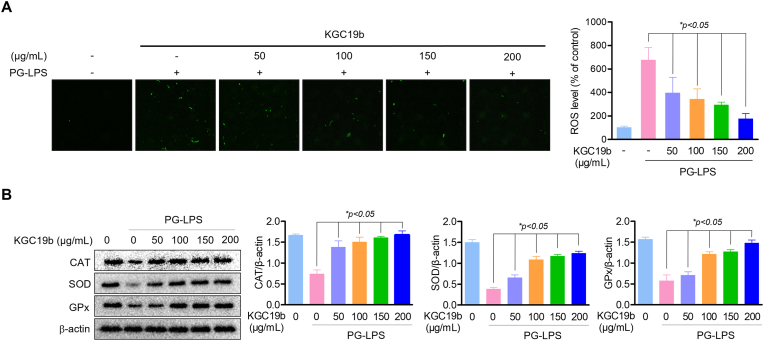
Reduction effect of KGC19b on oxidative stress markers in PG-LPS**-induced HPDL cells.** (A) Cells treated with indicated concentrations (50-200 μg/mL) of KGC19b and PG-LPS (1 μg/mL) using DCF-DA was investigated for ROS accumulation. (B) The level of SOD, CAT and GPx were measured by western blot analysis and normalized to that of β-actin. Data are expressed as mean ± SD (*n* = 3). *∗p* < 0.05, versus the only PG-LPS treated group.

Because oxidative stress acts as a critical mediator downstream of TLR4 signaling and contributes to osteogenic dysfunction, this study next examined whether KGC19b modulates intracellular ROS levels. PG-LPS stimulation induced a robust accumulation of intracellular ROS in HPDL cells, as assessed by DCF-DA fluorescence. KGC19b treatment significantly and dose-dependently reduced ROS accumulation under PG-LPS stimulated conditions (Fig. 3A). In parallel, KGC19b restored the expression of endogenous antioxidant enzymes, including Catalase (CAT), Superoxide Dismutase (SOD), and glutathione peroxidase (GPx) (Fig. 3B). These results clearly demonstrate that KGC19b mitigates PG-LPS–induced oxidative stress by reinforcing the cellular antioxidant defense system, thereby providing a redox-stable intracellular environment that supports osteogenic differentiation under inflammatory stress.

### 3.4 KGC19b exhibits antimicrobial activity against *P. gingivalis*

**Fig. 4:**
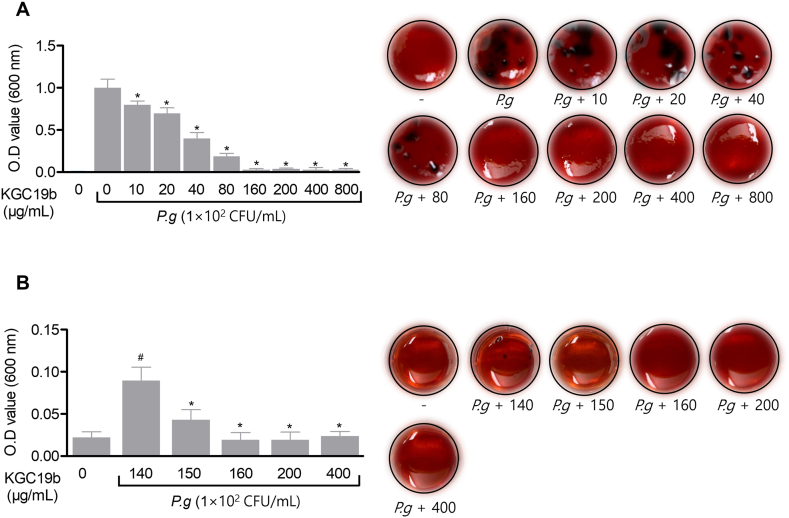
Antibacterial effects of KGC19b on *P. gingivalis*. (A) *P. gingivalis* (1 x 10^2^ CFU/mL) was cultured in TSA hemin menadione medium with various concentrations of KGC19b (10–800 μg/mL). After incubation, bacterial growth was assessed by measuring absorbance at 600 nm to determine the MIC. (B) After MIC analysis, the KGC19b (140–400 μg/mL) concentration group cultured in MBC analysis was diluted in series and absorbance was analyzed at 600 nm wavelengths using a microplate analyzer. Data are expressed as mean ± SD (*n* = 3). *∗p* < 0.05, versus the only PG-LPS treated group. ^*#*^*p* < 0.05, versus the control group.

In addition to its host-directed effects, this study further explored whether KGC19b directly targets the periodontal pathogen *Porphyromonas gingivalis*. Specifically, the Minimum Inhibitory Concentration (MIC) and Minimum Bactericidal Concentration (MBC) were determined to assess its antimicrobial activity. As shown in Fig. 4A, KGC19b inhibited the formation of black-pigmented colonies, a characteristic morphological feature of *P. gingivalis*, at approximately 160 μg/mL. Further evaluation of bactericidal activity revealed that KGC19b exerted clear antibacterial effects starting at 160 μg/mL, as confirmed by culture assays using concentrations above and below this threshold (Fig. 4B). These findings suggest that KGC19b may attenuate periodontal inflammation not only by modulating host inflammatory signaling but also by directly suppressing a key periodontal pathogen.

### 3.5 KGC19b attenuates periodontitis in a *PG*-LPS–induced *in vivo* model

**Fig. 5:**
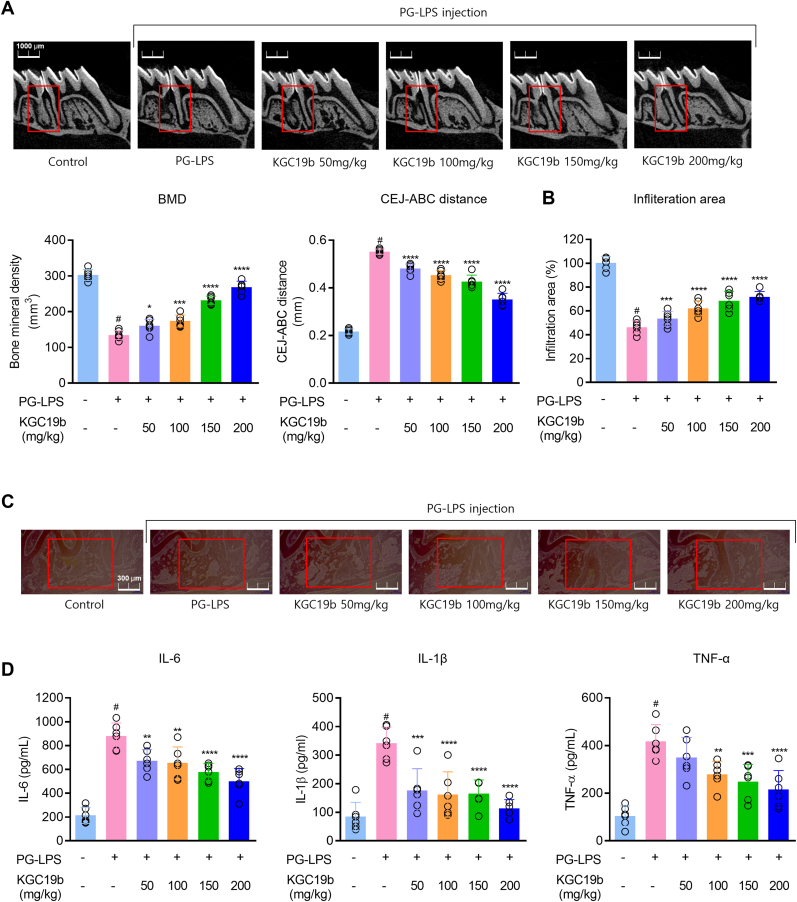
Inhibitory effects on periodontitis by oral administration of KGC19b in PG-LPS-induced *in vivo* model. (A) Images of alveolar bone newly formed by KGC19b were taken by micro-CT (3 mm) (Scale bar 1000 μM). (B, C) Periodontal tissue invasion by periodontitis was analyzed by H&E staining (Scale bar: 300 μM). (D) Measurement of pro-inflammatory cytokines in periodontitis-induced rat serum by PG-LPS induced using an ELISA kit. ^*#*^*p* < 0.05 vs control group; *∗p* < 0.05, *∗∗p* < 0.01, *∗∗∗p* < 0.001, *∗∗∗∗p* < 0.0001 vs. PG-LPS group. Red box: Region of Interest (ROI). Each group, *n* = 6, total of 36, Total experiment period: 14 days.

The protective efficacy of KGC19b was validated in a PG-LPS–induced acute periodontitis model. KGC19b was administered orally at doses ranging from 50 to 200 mg/kg, based on previously reported concentrations for ginseng, ginseng fruit extract, and red ginseng extract [50,51]. Following treatment, maxillary bone samples were collected and analyzed by micro-CT. As shown in Fig. 5A, KGC19b restored bone mineral density (BMD) and reduced cemento-enamel junction–alveolar bone crest (CEJ–ABC) distance, both of which were impaired by PG-LPS. Furthermore, H&E analysis revealed that KGC19b effectively restored or protected the area of inflammatory infiltration in PG-LPS-induced tissues. This was further confirmed by quantifying the area of infiltration, Therapeutic effects were demonstrated (Fig. 5B and C). Furthermore, KGC19b dose-dependently suppressed the production of pro-inflammatory cytokines, including TNF-α, IL-1β, and IL-6, and reduced inflammatory cell infiltration in periodontal tissues (Fig. 5D). Collectively, these findings demonstrate that KGC19b exerts a protective effect against PG-LPS–induced periodontitis *in vivo* by preserving alveolar bone and inhibiting inflammation.

### 3.6 KGC19b alleviates periodontitis in a ligature-induced *in vivo* model

**Fig. 6:**
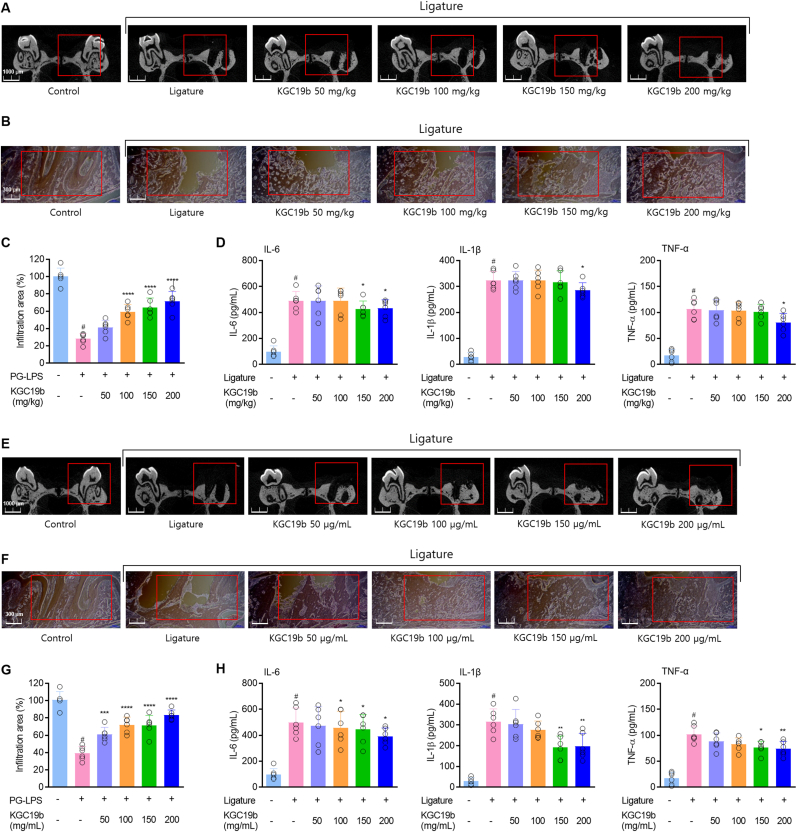
Inhibitory effects of KGC19b on ligature-induced periodontitis via oral and topical administration in an *in vivo* model. (A and E) Micro-computed tomography (Micro-CT) was performed to evaluate alveolar bone loss, and quantitative analysis was conducted using CTAn software (Scale bar: 1000 μM). (B, C and F, G) Histological analysis of periodontal tissue was performed using hematoxylin and eosin (H&E) staining (Scale bar: 300 μM). (D and H) Levels of pro-inflammatory cytokines in serum were measured by ELISA. ^*#*^*p* < 0.05 vs control group; *∗p* < 0.05, *∗∗p* < 0.01, *∗∗∗p* < 0.001, *∗∗∗∗p* < 0.0001 vs. ligature group. Red box: Region of Interest (ROI). Each group, *n* = 6, total of 36, Total experiment period: 14 days.

The therapeutic efficacy of KGC19b was evaluated through both oral administration and topical application in a ligature-induced periodontitis model. Periodontal socket regeneration and serum inflammatory cytokine levels were analyzed. Micro-CT analysis demonstrated that oral administration of KGC19b facilitated regeneration of ligature-induced periodontal tissue damage and significantly reduced alveolar bone loss (Fig. 6A). Similarly, topical application of KGC19b enhanced periodontal tissue recovery and was more effective than oral administration (Fig. 6E). Histological analysis using H&E staining further demonstrated that both treatment routes attenuated inflammatory infiltration and contributed to the preservation of periodontal tissue (Fig. 6B, C, F, G). Moreover, analysis of inflammatory cytokines showed that ligature upregulation of TNF-α, IL-1β, and IL-6 was dose-dependently suppressed by both oral and topical administration of KGC19b (Fig. 6D, H). Collectively, these findings suggest that KGC19b effectively suppresses chronic periodontal inflammation and bone destruction *in vivo*, supporting its therapeutic potential across distinct pathological contexts.

## 4 Discussion

The findings of this study support the concept that periodontitis should be regarded not merely as a localized bacterial infection, but as a chronic inflammatory disease driven by dysregulated PG-LPS–TLR4 signaling. PG-LPS acts as a primary etiological trigger that disrupts host immune homeostasis via TLR4, a critical pattern recognition receptor [15,16]. Our results demonstrate that activation of this pathway initiates a deleterious cascade characterized by excessive production of pro-inflammatory cytokines and accumulation of ROS. This persistent inflammatory milieu and oxidative stress collectively promote connective tissue degradation through upregulation of MMPs while simultaneously impairing osteoblast function and differentiation. By positioning the PG-LPS–TLR4 axis as a central upstream driver of both inflammation and osteogenic dysfunction, the present study provides a coherent mechanistic framework for the therapeutic effects of KGC19b. A defining feature of this research is the use of KGC19b, a ginseng fruit–derived extract, which distinguishes this study from the majority of ginseng research that has focused on root-derived compounds. Notably, KGC19b exhibited inhibitory effects on the growth of *P. gingivalis* (Fig. 4), suggesting an additional advantage in directly modulating the pathogenic oral microenvironment. Ginseng fruits are known to possess a distinct ginsenoside composition enriched in Re, Rd, and Rf, compared to ginseng roots [45]. In our previous work, individual ginsenosides derived from ginseng fruit were shown to exert cytoprotective effects through modulation of the HO-1/EGFR signaling axis [50,51]. Building upon these findings, the present study demonstrates that the ginseng fruit extract targets a more upstream and etiologically critical pathway the Pg-LPS–TLR4 axis thereby providing broader anti-inflammatory and osteoprotective effects in the context of periodontitis. Mechanistically, our results indicate that activation of TLR4 by PG-LPS serves as a critical upstream event that enhances intracellular oxidative stress and matrix-degrading activity in human periodontal ligament (HPDL) cells [29,60]. PG-LPS–induced TLR4 signaling promoted excessive ROS accumulation, which in turn increased the expression of matrix metalloproteinases, including MMP-1, and concomitantly suppressed osteogenic markers such as ALP and OCN (Fig. 2). Although further studies are needed to elucidate the mesenchymal stem cell-like phenotype in HPDL cells isolated during the osteogenic effector process, osteogenic differentiation is a HPDL cell property not seen in epithelial cells, strongly supporting the mesenchymal nature of the cell population. These findings demonstrate that TLR4 activation contributes not only to inflammatory tissue degradation but also to the impairment of osteogenic function in HPDL cells, which possess inherent mesenchymal characteristics and osteogenic differentiation capacity. Through upregulation of endogenous antioxidant enzymes (SOD, CAT, and GPx), KGC19b established a redox-balanced cellular environment that protected osteoblasts from inflammation-induced damage and preserved their differentiation capacity under PG-LPS challenge (Fig. 3). This study employed two mechanistically distinct *in vivo* models of periodontitis [[30], [31], [32], [33], [34], [35], [36], [37], [38]]. The consistent protective effects of KGC19b observed in both Pg-LPS–induced and ligature-induced models indicate that its therapeutic efficacy is maintained across different pathological contexts [39]. These *in vivo* outcomes support the notion that suppression of TLR4-associated inflammatory signaling by KGC19b translates into meaningful preservation of periodontal tissue integrity and alveolar bone. Despite these promising results, several limitations should be acknowledged. Although this study elucidates the role of TLR4-associated inflammatory and oxidative signaling in mediating the effects of KGC19b, several limitations should be acknowledged. In particular, the downstream signaling pathways and potential crosstalk with other innate immune receptors remain to be explored. The present findings provide important mechanistic and biological insights into the therapeutic potential of KGC19b. In conclusion, this study demonstrates that KGC19b alleviates Pg-LPS–induced periodontitis by suppressing TLR4-mediated inflammatory and oxidative stress signaling while preserving osteogenic function. Taken together, these results position ginseng fruit–derived extracts as promising candidates for the development of novel therapeutic strategies targeting both inflammation and bone loss in periodontitis.

## 5 Conclusions

In this study, KGC19b emerges as a promising therapeutic candidate for the treatment of periodontitis, owing to its unique \ability to modulate both inflammatory and regenerative pathways. Future studies should aim to elucidate the detailed molecular mechanisms of KGC19b, particularly its role in bone remodeling, and conduct clinical trials to assess its safety and efficacy in human patients. These efforts will be essential to establish KGC19b as a novel dual-action therapy for periodontitis and other inflammatory bone diseases.

## Declaration of competing interest

The authors declare that they have no conflict of interests.

## Data Availability

The authors declare no known financial conflicts of interest or personal relationships that may have influenced the findings reported in this paper.
