# Supplementary material for: Alleviating effects of KGC19b on periodontal inflammation through anti-inflammatory and induction of osteogenic differentiation in in vitro and in vivo experimental models
Source: J Ginseng Res. 2026 Feb 21;50(3):101014. doi: 10.1016/j.jgr.2026.101014 (PMC13149898; doi:10.1016/j.jgr.2026.101014)
Supplement: Multimedia component 1 [file mmc1.docx]

**Supplemental data**

Da Eun Lee^1†^, Eun-Nam Kim^1†^, Nguyen Minh Trang^1^, Jong Han Kim^2^, Gi-Bang Koo^2^, Hee Jung You^3^, Gil-Saeng Jeong^1*^

^1^College of Pharmacy, Chungnam National University, Daejeon, 34134, Republic of Korea.

^2^Laboratory of Efficacy Research, Korea Ginseng Corporation, Gyeonggi-do,13480, Republic of Korea.

^3^Laboratory of Standards and Fundamental Research, Korea Ginseng Corporation, Gyeonggi-do, 13480, Republic of Korea.

daeunlee7037@gmail.com; enkim@cnu.ac.kr; ngminhtrang52@gmail.com; gsjeong@cnu.ac.kr; bellone@kgc.co.kr; 20170068@kgc.co.kr; 20030078@kgc.co.kr.

*Correspondences:

Professor Gil-Saeng Jeong, College of Pharmacy, Chungnam National University, Daejeon 34134, Republic of Korea. E-mail: gsjeong@cnu.ac.kr

^†^Da Eun Lee and Eun-Nam Kim contributed equally to the article.

**Table S1**

**Primer sequences for real-time PCR analysis**

| Targets | Sequences (5’ – 3’) | |
| --- | --- | --- |
| *gapdh* | Forward | GTCTCCTCTGACTTCAACAGCG |
|  | Reverse | ACCACCCTGTTGCTGTAGCCAA |
| *il-6* | Forward | AGACAGCCACTCACCTCTTCAG |
|  | Reverse | TTCTGCCAGTGCCTCTTTGCTG |
| *il-1β* | Forward | CTGTACCTGTCCTGCGTGTT |
|  | Reverse | GGGAACTGGGCAGACTCAAA |
| *tnf-α* | Forward | GCCTCTTCTCCTTCCTGATCGT |
|  | Reverse | TGAGGGTTTGCTACAACATGGG |
| *alp*  *ocn* | Forward  Reverse | GATGGGATGGGGGTGTCTAC  GTCCATGGCCAGGGGTATCT |
|  | Forward  Reverse | CGCTACCTGTATCAATGGCTGG  CTCCTGAAAGCCGATGTGGTCA |
| *timp1* | Forward  Reverse | GGAGAGTGTCTGCGGATACTTC  GCAGGTAGTGATGTGCAAGAGTC |
| *mmp1* | Forward  Reverse | ATGAAGCAGCCCAGATGTGGAG  TGGTCCACATCTGCTCTTGGCA |

**Figure S1.**


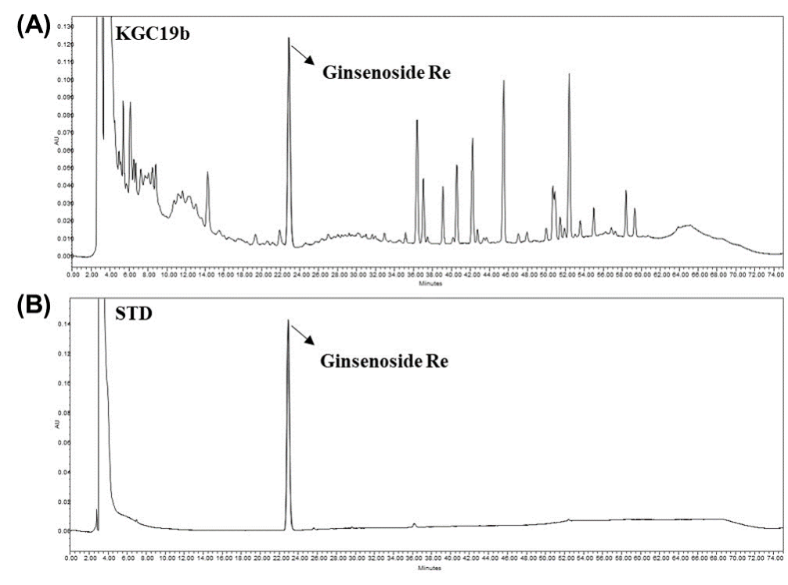


Fig. S1. HPLC chromatograms of KGC19b (A) and Ginsenoside Re STD (B) using the analysis procedure for ginsenoside.

**Figure S2.**

**
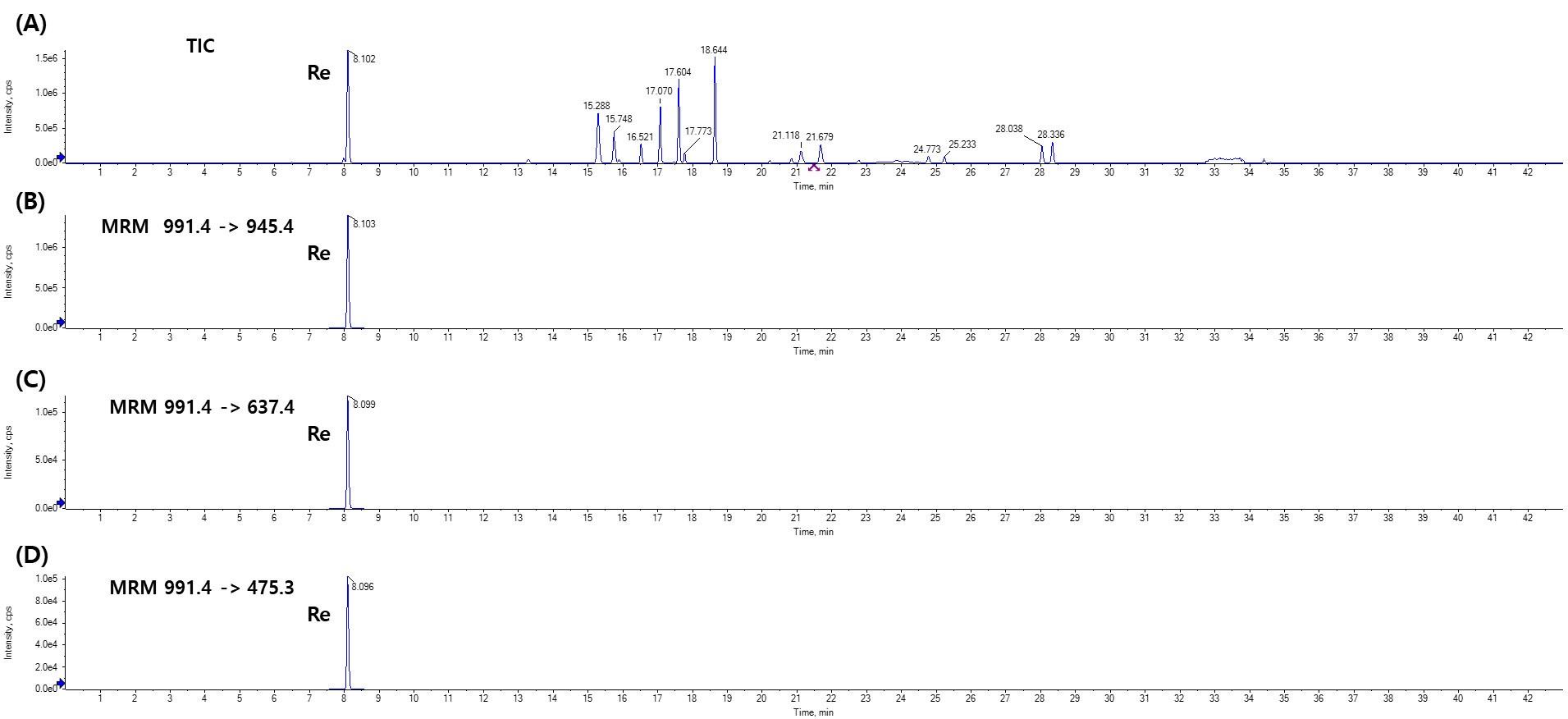
**

Fig. S2. Total ion chromatogram (TIC) of KGC19b (A), Proton ion *m/z* 945.4 (B), Proton ion *m/z* 637.4 (C), Proton ion *m/z* 475.3 (D) of ginsenoside Re.
